# Coexistence of Pulmonary Thromboembolism, Pulmonary Tuberculosis and Granulomatosis with Polyangiitis: A flimsy triple dribble

**DOI:** 10.18295/squmj.3.2024.021

**Published:** 2024-08-29

**Authors:** Sai T. Pavirala, Alkesh Khurana, Kirti Kadian, Abhishek Goyal

**Affiliations:** Department of Pulmonary Medicine, AIIMS Bhopal, Madhya Pradesh, India

**Keywords:** Granulomatosis with Polyangiitis, Pulmonary Tuberculosis, Deep Venous Thrombosis, Vasculitis, c-ANCA, Case Report, India

## Abstract

Granulomatosis with polyangiitis (GPA) is a rare autoimmune disease with multi-system involvement. It involves the upper respiratory tract, lungs and kidneys. A 36-year-old female patient presented to a tertiary care referral hospital in Central India in 2023 with complaints of low-grade fever, dry cough and loss of appetite initially followed by dyspnoea, purpuric skin lesions, right lower limb swelling with pain and redness. Her chest radiograph revealed right upper lobe cavitary lesion with consolidation in the right lower lobe. Mycobacterium tuberculosis was detected in sputum and broncho alveolar lavage via cartridge based nucleic acid amplification assay. Later, computed tomography pulmonary angiography revealed bilateral pulmonary artery thromboembolism. Furthermore, her cytoplasmic-antineutrophil cytoplasmic antibody test was positive, serum creatinine was rising, urine microscopy had red cell casts and lower limb venous doppler revealed deep venous thrombosis. Histopathological examination of the skin lesion revealed vasculitis. Based on these findings, the patient was diagnosed with GPA. The patient improved with pulse steroids, cyclophosphamide, anticoagulants and anti-tuberculous therapy.

Granulomatosis with polyangiitis (GPA) formerly known as Wegner’s granulomatosis is a systemic vasculitis involving small vessels predominantly.^1^ In countries with high prevalence of tuberculosis (TB), the diagnosis can be challenging as the presentation of GPA is heterogenous and can mimic TB due to its clinico-radiological overlap.^2^ In addition, immunosuppressive therapy, which is the main-stay of treatment for GPA can also lead to an increased risk of infections such as TB. We present an extremely rare case of GPA who, at presentation, had pulmonary tuberculosis as well as pulmonary thromboembolism. Whether there was an increased predisposition of one disease because of the other or an extremely rare coincidence of all 3 diseases occurring together remains debatable.

## Case Report

**Figure 1:**
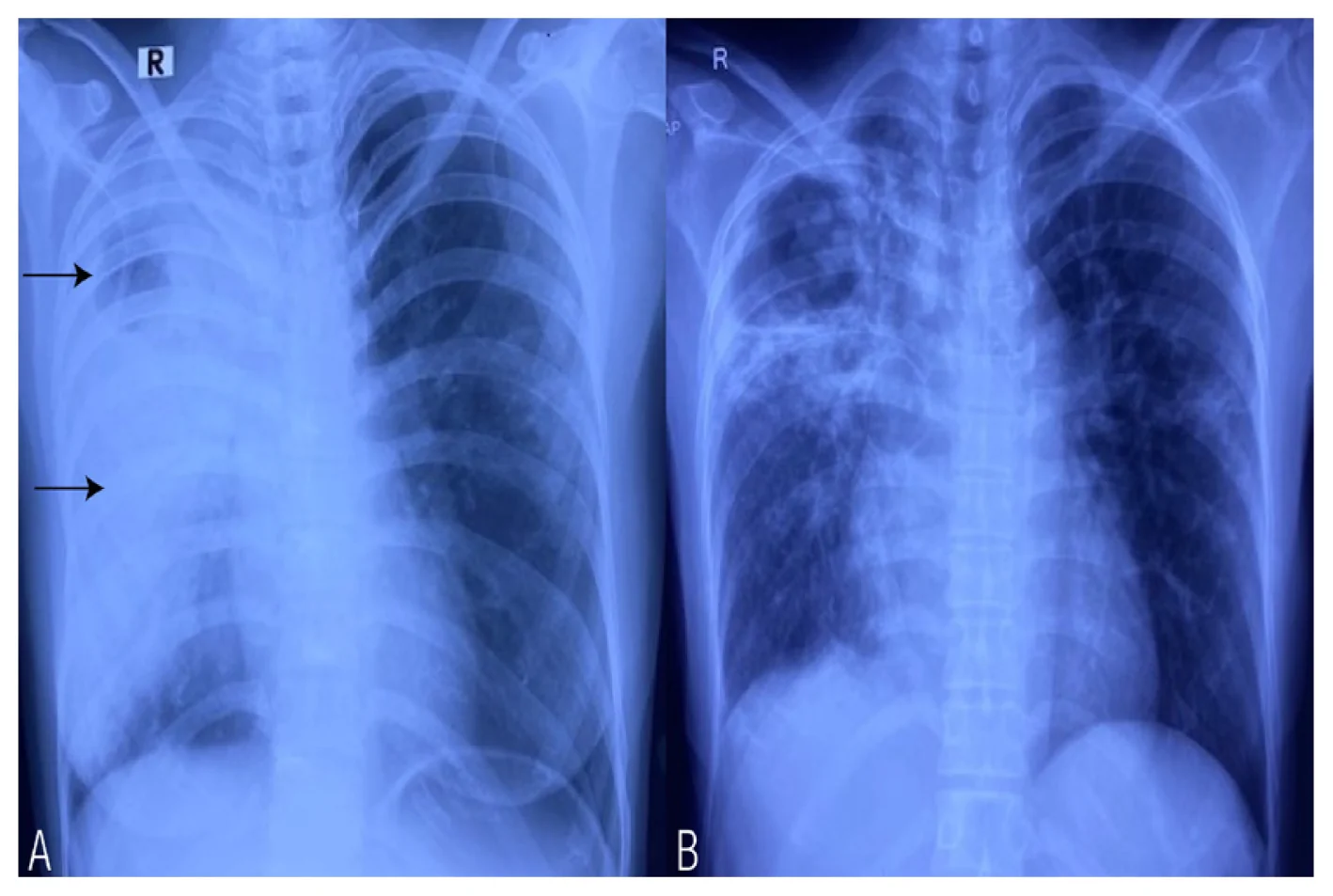
**A**: Chest radiograph showing a cavity in the right upper lobe (upper arrow) along with consolidation in right upper and lower lobes (lower arrow). **B**: Follow-up chest radiograph showing significant resolution of the consolidation.

A 36-year-old female patient, a house-wife without any obvious risk factors and co-morbidities, presented to a tertiary care referral hospital in Central India in 2023 with complaints of low-grade fever, dry cough and loss of appetite for 1 month. On evaluation by a general practitioner, she was suspected as having pulmonary TB as her chest radiograph showed a cavity in the right upper lobe along with consolidation in right upper and lower lobes [Figure 1A]. This was then confirmed by sputum examination for acid fast bacillus (AFB) by cartridge based nucleic acid amplification test (CBNAAT), which was positive. She was hence started on anti-tuberculous therapy (ATT). However, there was not much improvement clinically even after 1 month of starting ATT and she developed shortness of breath, purpuric skin lesions, epistaxis and also accompanying right lower limb swelling with pain and redness. On initial examination her blood pressure was 138/88 mmHg, oxygen saturation was 88% at room air and pulse rate was 92 beats/minute. Her haemogram test and serum electrolytes were normal but urine routine and microscopy showed red cell casts, blood urea nitrogen was 16.07 mmol/L, plasma creatine was 167.2 μmol/L (which gradually increased to 387.2 μmol/L) and elevated D-dimer levels (2.3 FEU/L). Urine and blood cultures were found to be normal.

**Figure 2:**
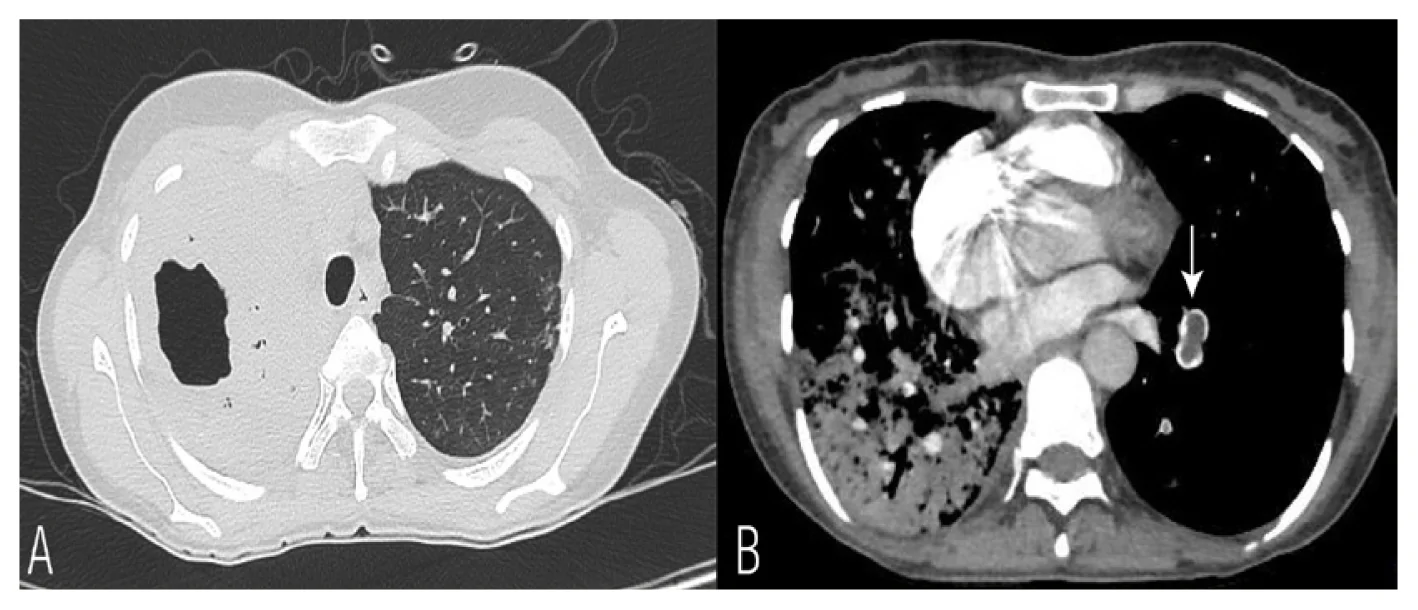
**A**: Parenchymal window of computed tomography (CT)thorax showing dense consolidation in right hemithorax with a cavity in right upper lobe. **B**: CT pulmonary angiogram image revealing hypodense thrombus in the arterial branch of left lower lobe (vertical arrow) highly suggestive of pulmonary thromboembolism.

**Figure 3:**
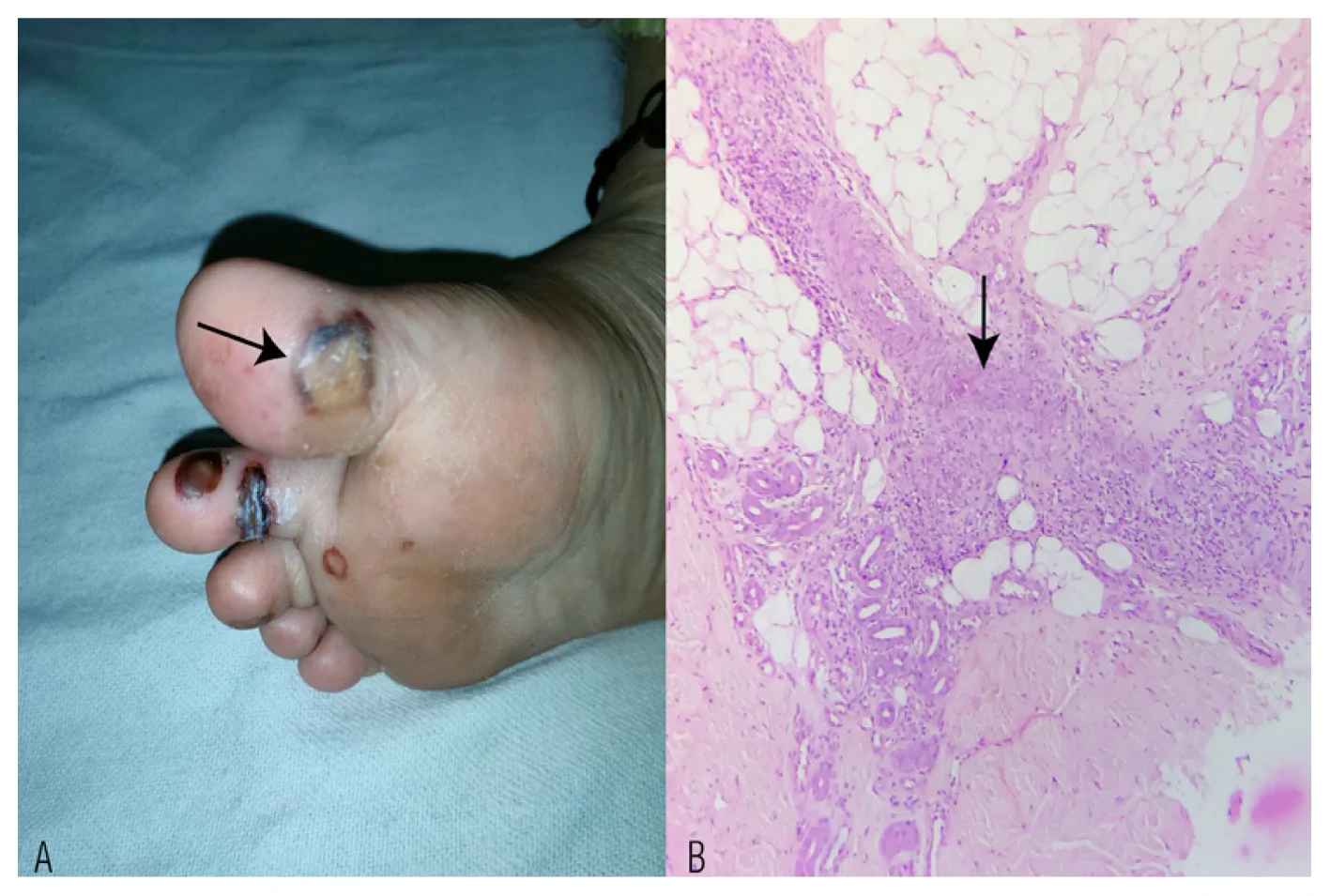
**A**: Photograph showing haemorrhagic vesicles with perilesional purpura and erythema over the sole region (arrow). **B**: Haematoxylin and eosin stain of subcutaneous tissue at ×100 magnification showing medium-sized vessels infiltrated by histiocytes in aggregates, lymphocytes and a few neutrophils. The vessel wall shows focal fibrinoid necrosis (arrow).

Arterial and venous colour doppler of bilateral limbs revealed long segment hypoechoic thrombus in right saphenous and popliteal vein with no arterial thrombus. 2-dimensional echocardiography showed dilated right atrium, right ventricle internal diameter (RVID = 3.23 cm) with peak systolic right ventricular pressure of 56 mmHg, mild tricuspid regurgitation with normal right ventricle and left ventricle systolic function. On further evaluation, computed tomography pulmonary angiography (CTPA) was done. Parenchymal window showed dense consolidation in right hemithorax with a cavity in right upper lobe and angiogram images revealed hypodense thrombus in the lumen of segmental arteries of right lower lobe and left lower lobar artery suggestive of pulmonary thromboembolism [Figure 2]. Autoimmune work-up revealed negative antinuclear antibodies (ANA) profile whilst cytoplasmic-antineutrophil cytoplasmic antibody (c-ANCA) was strongly positive (>200 RU/mL, biological reference: <20, done via immunofluorescence). Perinuclear-ANCA, C4 and C3 were all negative. Punch biopsy from the purpuric skin lesions showed vasculitis consistent with GPA [Figure 3]. Flexible bronchoscopy revealed no obvious endobronchial growth as such, but bronchoalveolar lavage (BAL) for AFB and CBNAAT was positive for mycobacterium TB. Diagnosis of GPA was made based on serology, involvement of respiratory tract, haematuria and skin biopsy.

Since GPA with deep venous thrombosis and pulmonary TBwere diagnosed almost simultaneously, a combined treatment for GPA, TB and deep venous thrombosis was promptly started at the same time, to avoid further worsening of patient’s clinical condition. The patient was treated with pulse methylprednisolone 1 g for 3 days followed by gradual tapering of steroids along with cyclophosphamide. Anti-TB treatment was started along with systemic anticoagulation (initially started with heparin and gradually switched to rivaroxaban). The patient improved significantly over the subsequent weeks and is under regular follow-up [Figure 1B].

Patient consent was obtained for the publication of this case.

## Discussion

The diagnosis of GPA is based on a combination of various clinical manifestations of a systemic disease suggestive of vasculitis; positive ANCA serology and histological evidence of necrotising vasculitis, necrotising glomerulonephritis or granulomatous inflammation from a relevant organ biopsy, such as the skin, lung or kidney.^2^ The diagnosis of concomitant GPA and TB is challenging because firstly clinical features of TB and GPA are overlapping, secondly despite considerable specificity of c-ANCA in GPA, c-ANCA levels have occasionally been reported to be raised in patients with TB.^3^ Both these aetiologies coexisted in the current patient as on one hand, AFB was detected twice in sputum as well as BAL, and on the other hand, a positive c-ANCA, vasculitis on skin histopathology and dramatic response of lung consolidation to steroids confirmed the presence of GPA.^4^

Both TB and ANCA associated vasculitis can lead to a hypercoagulable state and lead to an increased incidence of venous thromboembolic (VTE) diseases.^5^ Patients appear to be particularly at risk especially during active periods of inflammation.^6^ Occasional detection of ANCA in TB may also suggest triggering of an autoimmune reaction with mycobacterium TB as the inciting antigen. Although one can debate these manifestations being bracketed under one broad spectrum of TB, the authors would prefer to label GPA as an independent occurrence because of the combined presence of c-ANCA, vasculitis on skin biopsy, response to steroids and lack of drug induced lupus/ANA.

The Wegener’s Clinical Occurrence of Thrombosis study recruited 180 patients during active periods of disease. The reported incidence of VTE was 7.0 per 100 person-years (95% confidence interval: 4.0–11.4).^7^ In a case report published in Iran, a 28-year-old male was diagnosed with both TB and GPA and was hence started on both immunosuppressants and ATT; the patient eventually developed cerebral venous thrombosis which was treated with anticoagulation medication. In another case published by Khilani *et al*., a patient was initially started with ATT based on clinico-radiological features but eventually turned out to be Wegners following detection of vasculitis and c-ANCA.^4^ The current patient was also found to have both TB and GPA, but with deep venous thrombosis at the time of presentation which is a very rare entity.^8^

The complexity of the possible inter-relationships between the disease entities enables more than one hypothesis to be possible; it is virtually impossible to determine which one leads to the other. But the simultaneous detection of 3 entities, which can otherwise exist independently also, makes this case worthwhile and intriguing.

Co-existing diagnosis of these 3 entities is a challenge to manage. This is because immunosuppressants such as steroids and cyclophosphamide is the gold standard treatment in Wegners, while these may increase the severity of TB. However, treatment of Wegners is warranted to reduce mortality and morbidity in due course. At the same time, Wegners leads to progressive renal disease which leads to change in the ATT regimen as per the renal parameters.

Though immunosuppressive therapy is relatively contraindicated in patients with active TB, untreated GPA might be life-threatening. Moreover, combined treatments for both vasculitis and TB shows positive patient response, according to published case reports.^9^^,^^10^

## Conclusion

To the best of the authors’ knowledge, this is the first case report presenting a coexisting diagnosis of GPA, pulmonary TB, deep venous thrombosis and pulmonary thromboembolism. Therefore, clinicians should be aware of potential multiple differential diagnoses when considering diagnosis and treatment.
